# Thumb reconstruction by “on-top-plasty” of the long finger

**DOI:** 10.1080/23320885.2021.1935965

**Published:** 2021-07-09

**Authors:** Mark A. Greyson, Sarah Kinsley, Simon G. Talbot

**Affiliations:** Division of Plastic SurgeryDivision of Plastic and Reconstructive Surgery, Brigham and Women’s Hospital, Harvard Medical School, Boston, MA, USA

**Keywords:** Thumb, reconstruction, amputation, pollicization, ‘on-top’ plasty

## Abstract

A 61 year old man sustained electrical burns to hands requiring amputation of the right thumb and index finger at the metacarpophalangeal level. The thumb was reconstructed by means of on-top-plasty of the long finger. This is a reliable and safe technique in selected patients with severe, traumatic hand injuries.

## Introduction

After initial patient stabilization and exclusion of life-threatening injuries, the goal of the surgeon in a mutilating hand burn or trauma is to reconstruct a hand that is maximally useful. While a devastating injury can at first seem overwhelming, focusing on a few basic principles can help guide the initial management of these injuries and set the stage for future interventions. In a very basic sense, the surgeon should maintain an initial focus on preserving limb and digital length [1], with prioritization and attention to the thumb [2]. Additionally, the surgeon must decide which tissues require debridement, which can be revascularized, and always be mindful of those tissues which could be used as ‘spare parts’ for future constructs [3]. A recognition of the fundamental requirements of a working hand including stability, functional elements such as pinch and grip, and sensation should be all maintained in equipoise [4–6].

With regard to thumb reconstruction after amputation, there are a multitude of options available including distraction lengthening, free toe transfer, axial or free osteoplastic techniques, osseointegrated prostheses, pollicization, or transposition of an adjacent digit as a stable post, otherwise known as ‘on-top-plasty’. The appropriate operation is dependent on multiple variables including patient-specific factors such as age, occupation, and medical co-morbidities, and injury-related factors, most importantly, amputation level and zone of injury.

The techniques of transferring adjacent digits to the thumb position after mutilating hand trauma have their genesis in the decades after World War I, and were further popularized by J. William Littler [7]. The term ‘on-top-plasty’ was first coined by Henrik Søiland of Stavanger, Norway in 1961 in describing his experience with two patients who had intact thumbs but amputations of all fingers through the proximal phalanges, a common industrial injury pattern at the time. In these cases he transposed the remnant of the small finger to the ring finger position to achieve greater length and function [8]. This concept is a powerful one, and affords the transfer of any finger remnant to any other position on the hand, limited only by the arc of rotation of the neurovascular pedicle [9]. The use of an index finger remnant for such a transposition to the thumb position has been well described, however there is a paucity of literature describing transposition of other digits for thumb reconstruction [10,11]. In this report, we describe the use of a long finger remnant transposed to the thumb position to restore length and achieve tip pinch.

## Case presentation

**Figure 1.**
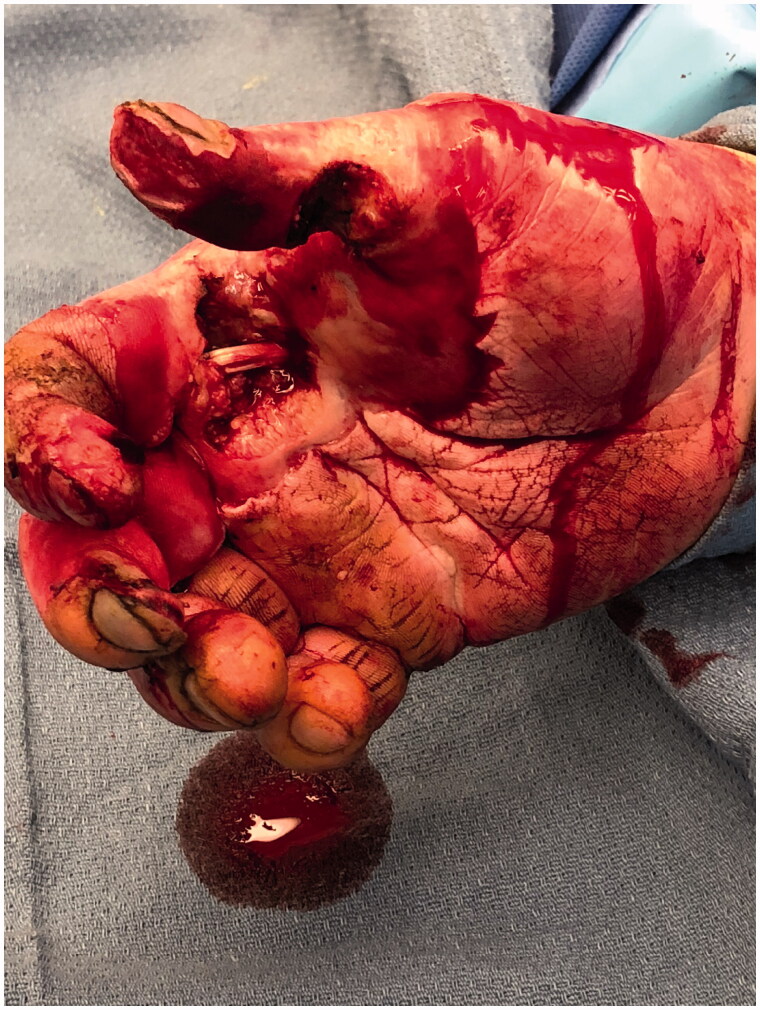
Oblique view of the blast injury resulting in dysvascular right thumb and flexor tendon injury to the index finger.

**Figure 2.**
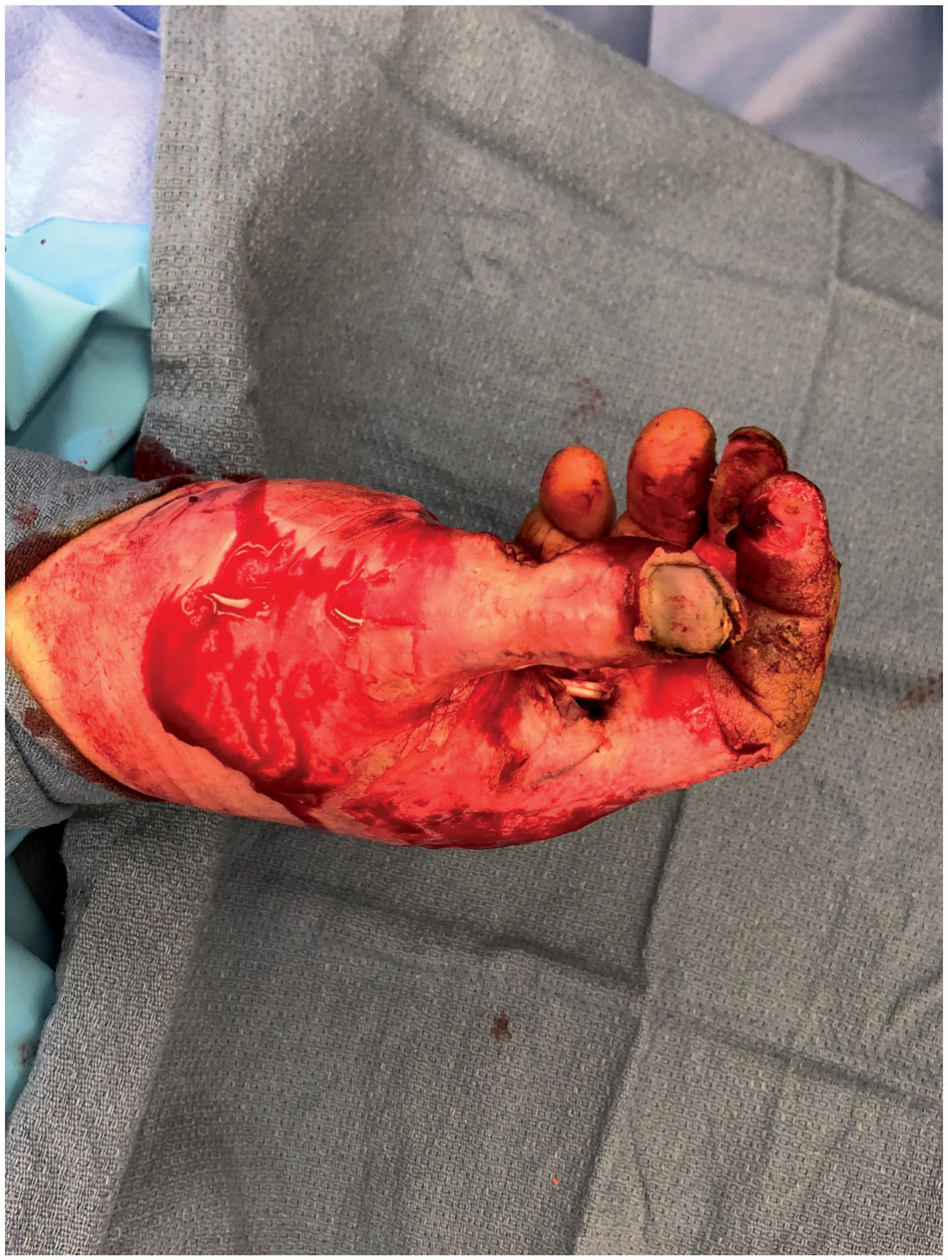
Lateral view of the blast injury.

**Figure 3.**
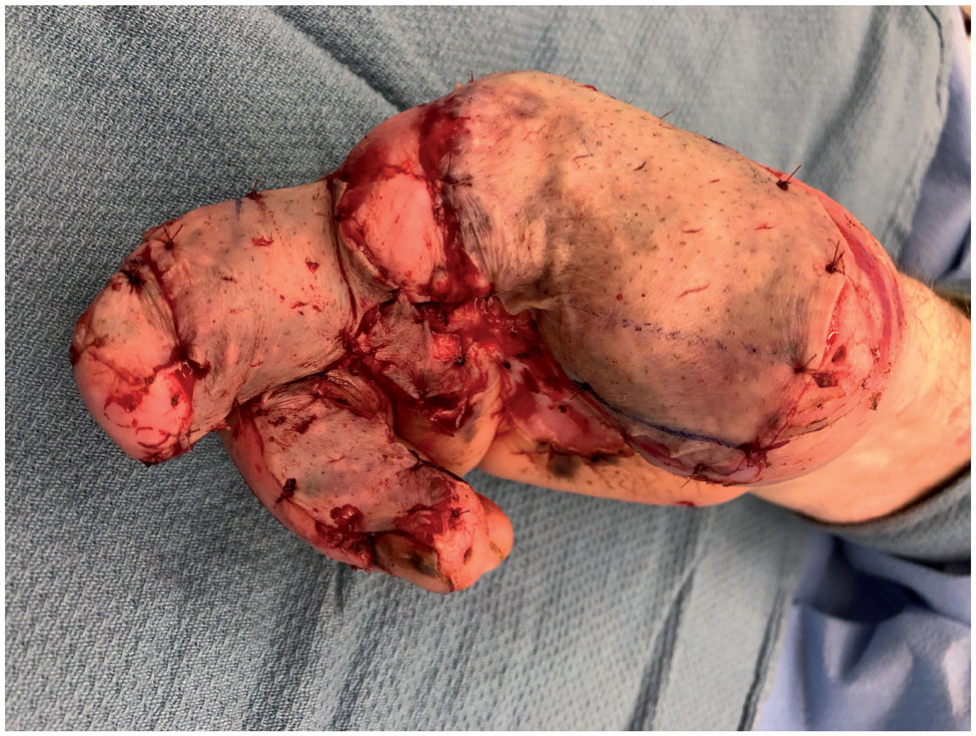
Wound appearance following split thickness skin grafting in the sub-acute period.

We present the case of a 61-year-old right hand dominant man with a history of type 2 diabetes and regular marijuana use, who presented with electrical burns to his chest and bilateral hands after a craft activity. Specifically, he was using a microwave oven electrical circuit to etch wood in a process called fractal wood burning. His right hand injuries included predominantly full-thickness burns to the thumb, index, and long fingers, a dysvascular thumb beyond the interphalangeal joint (IP) and blast injuries causing segmental loss of the flexor digitorum superficialis (FDS) and flexor digitorum profundus (FDP) of the index finger (Figures 1 and 2). Initial management included conservative tangential excision and serial debridement of clearly devitalized soft tissue and repair of the FDP tendon of the right index finger. Eight days later at a second look operation, progressive necrosis of the affected soft tissue was apparent, with destruction of the radial digital bundle to the index finger, rendering the finger functionally useless. Similarly, necrosis of the thumb had progressed proximally to the level of the metacarpophalangeal joint (MP). This necessitated amputation of the right thumb and index finger at the MP joint and the long finger at the distal IP level. The soft tissue of the palm was reconstructed with a filet flap from the index finger based on the intact ulnar digital bundle. Further soft tissue coverage of the right hand was achieved with split thickness skin grafting several weeks later (Figure 3).

**Figure 4.**
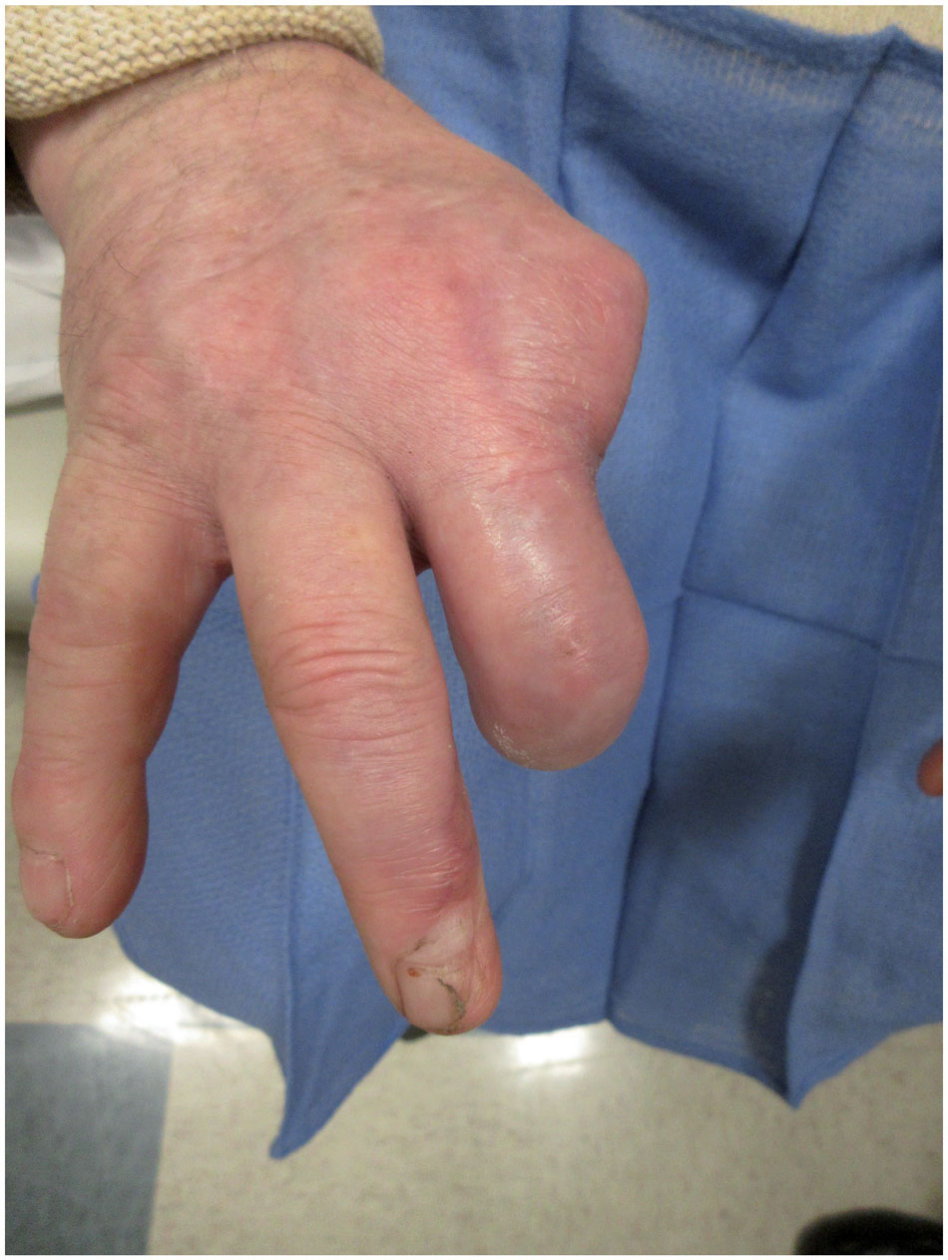
PA view of the hand 18 months following the original injury.

**Figure 5.**
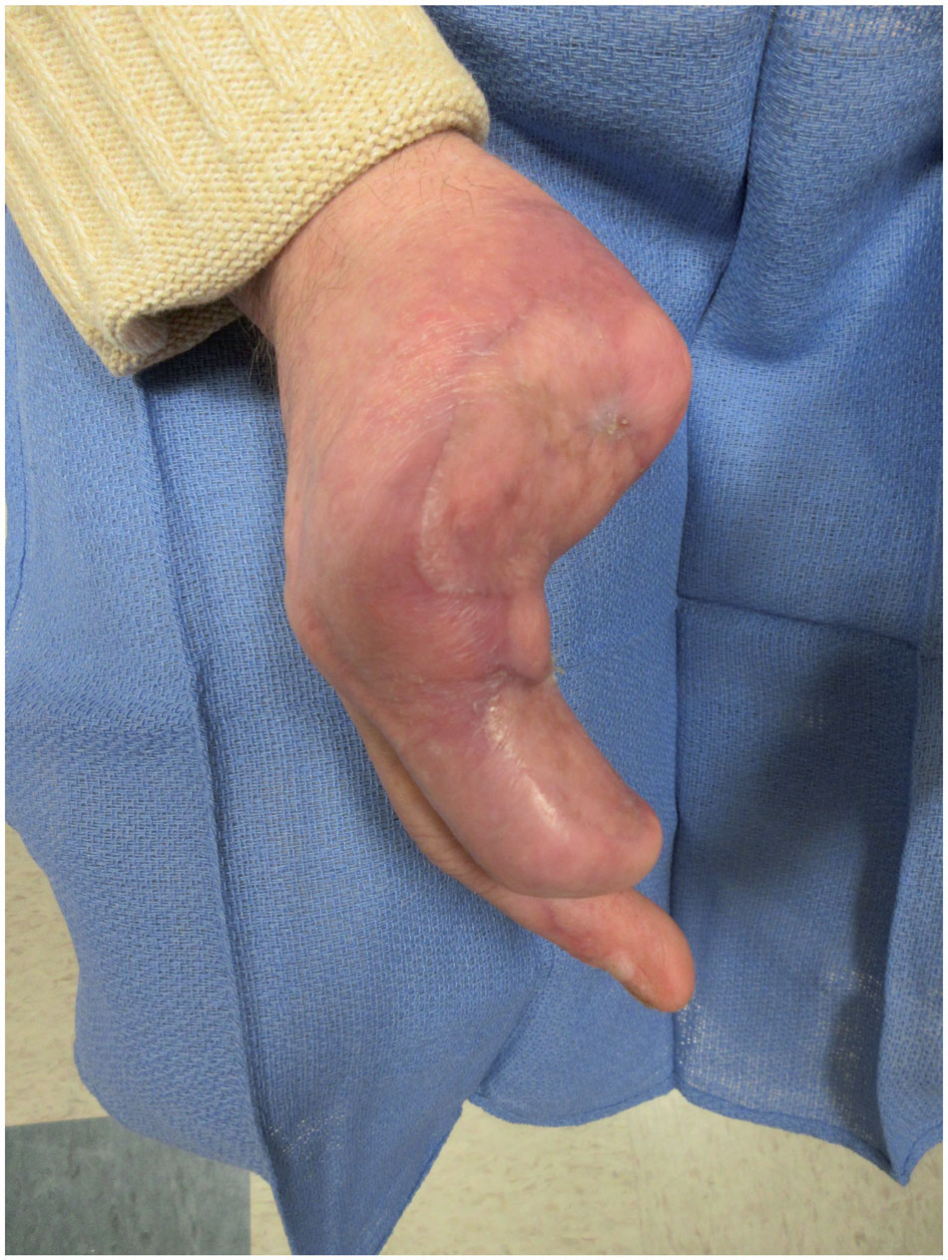
Lateral view of the hand 18 months following the original injury.

**Figure 6.**
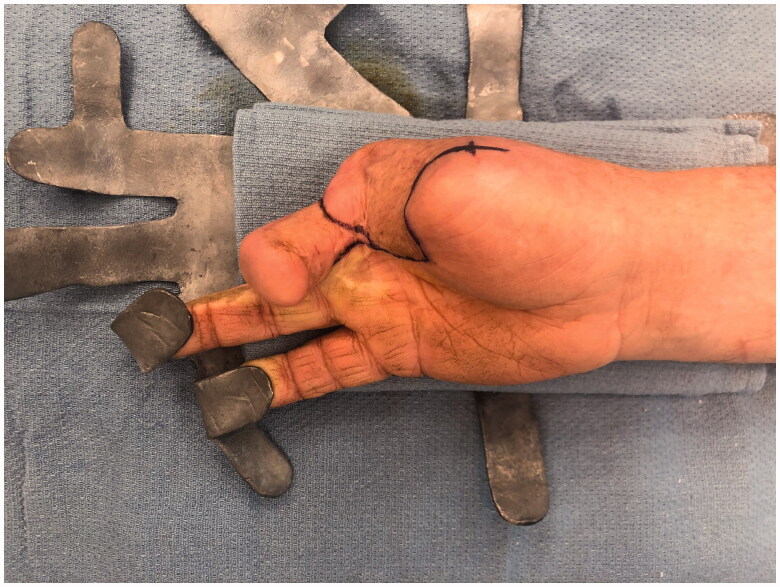
Preoperative markings for “on-top”-plasty of the long finger to the thumb position - PA view.

**Figure 7.**
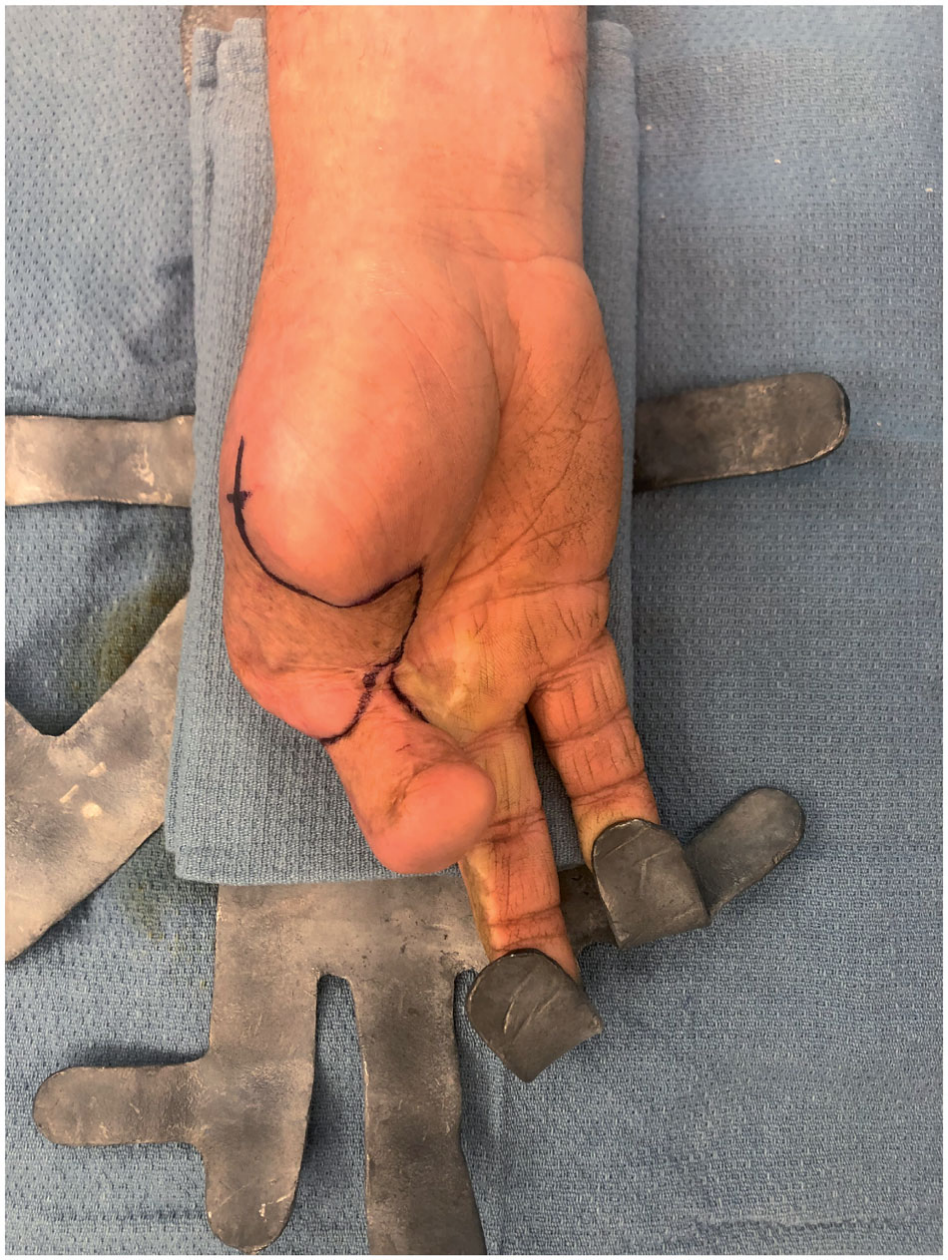
Preoperative markings for “on-top”-plasty of the long finger to the thumb position - PA bird's-eye view.

**Figure 8.**
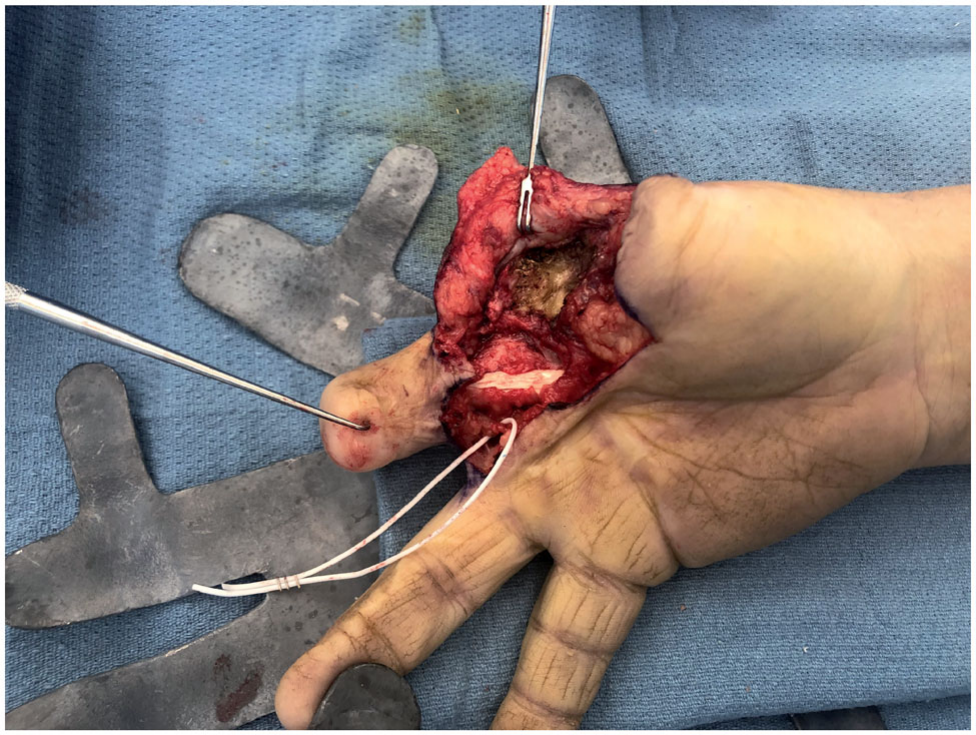
Dissection of the long finger on the ulnar digital neurovascular bundle.

**Figure 9.**
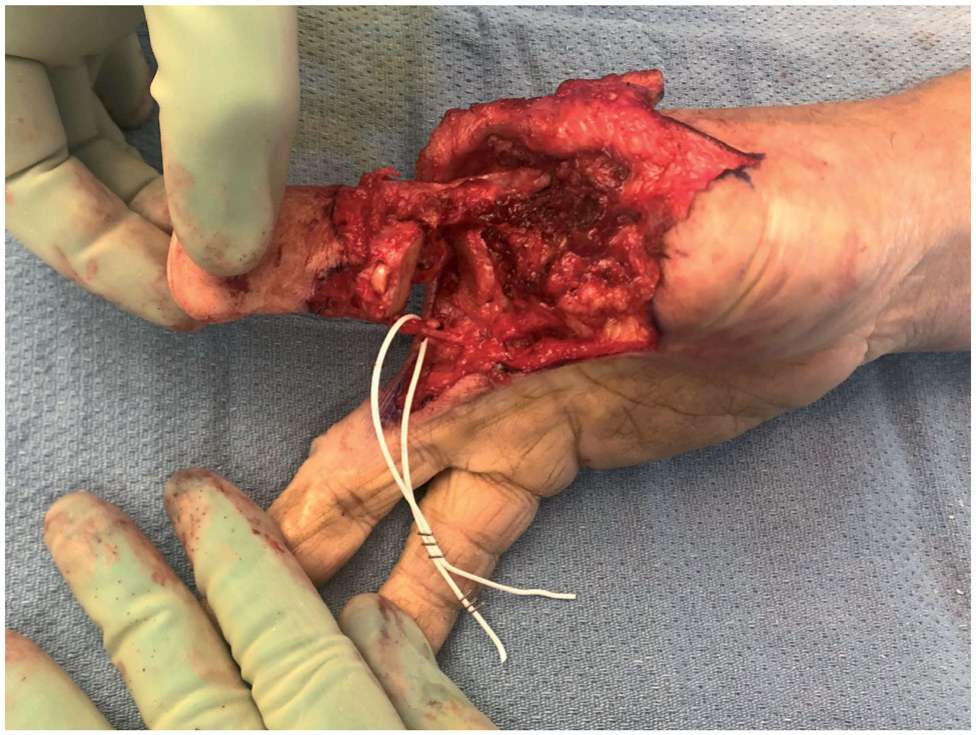
Partial ray resection of the long finger metacarpal and transposition of the remaining digit on the ulnar neurovascular pedicle.

**Figure 10.**
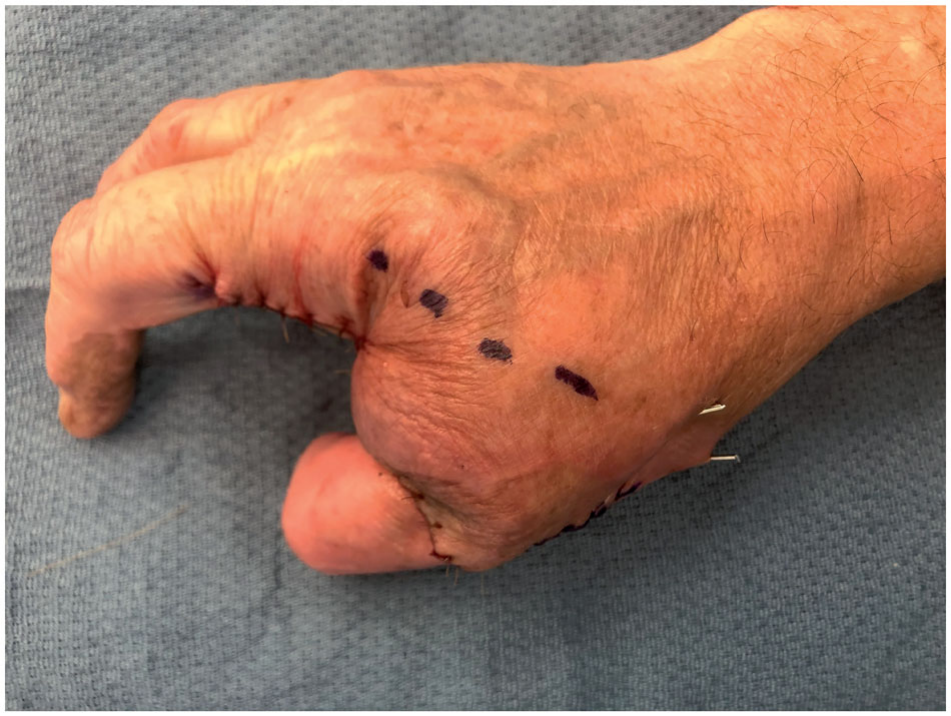
Post-operative appearance following Kirschner wire fixation of the long finger onto the thumb metacarpal.

**Figure 11.**
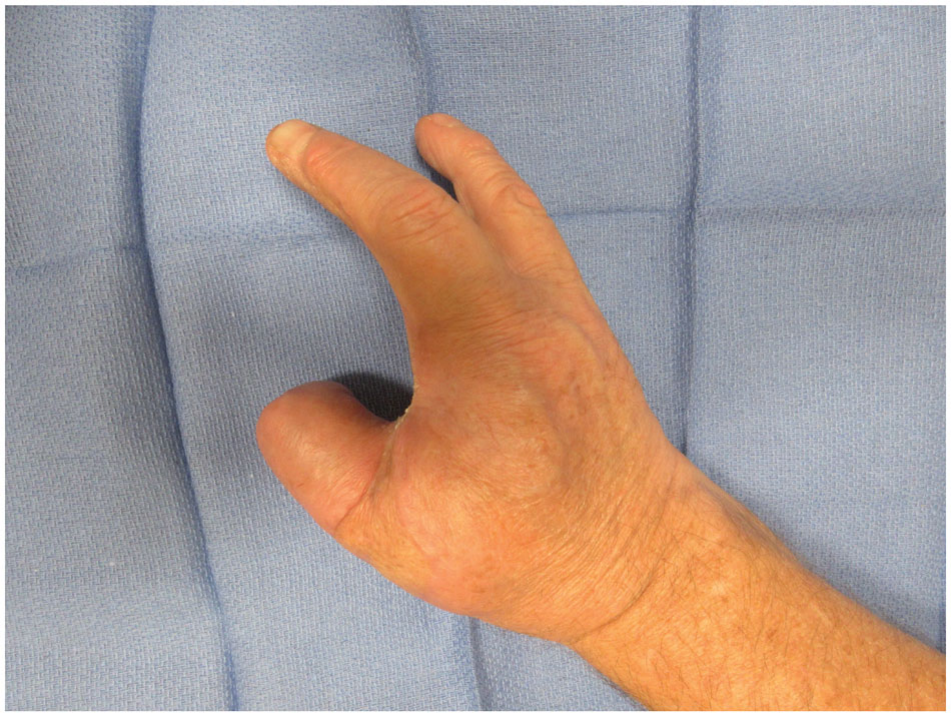
3 month post-operative appearance - oblique view of the hand.

**Figure 12.**
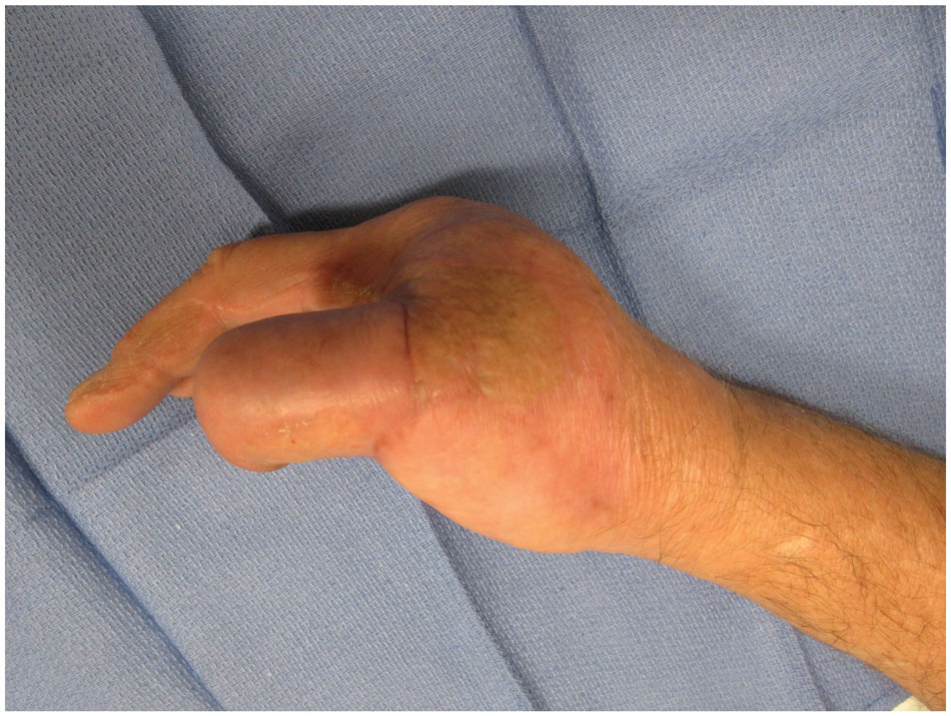
3 month post-operative appearance - lateral view of the hand.

**Figure 13.**
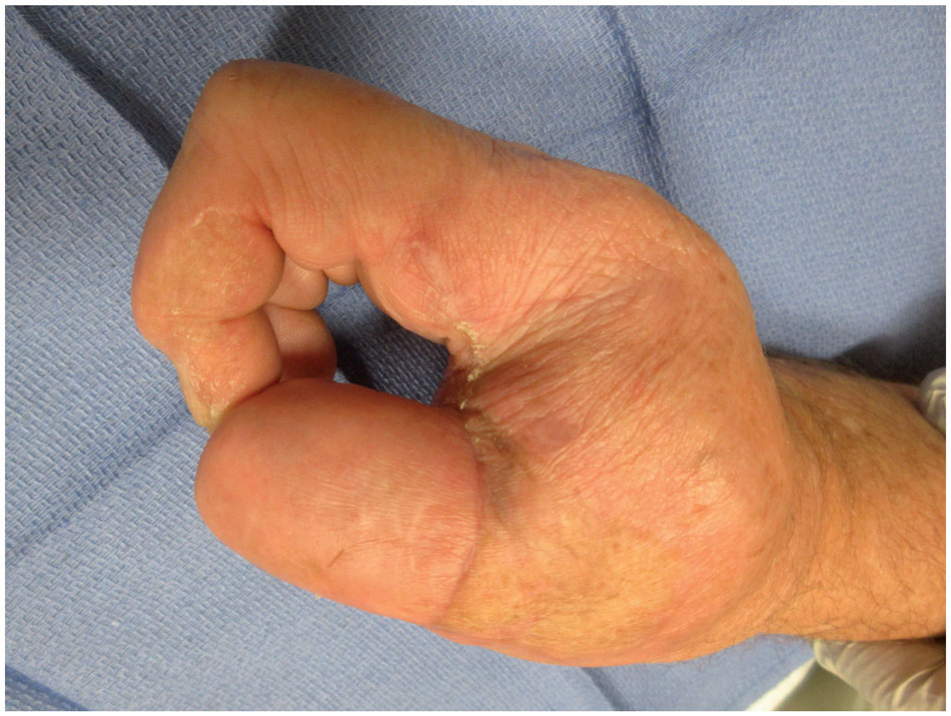
3 month post-operative appearance - PA view of the reconstructed thumb.

**Figure 14.**
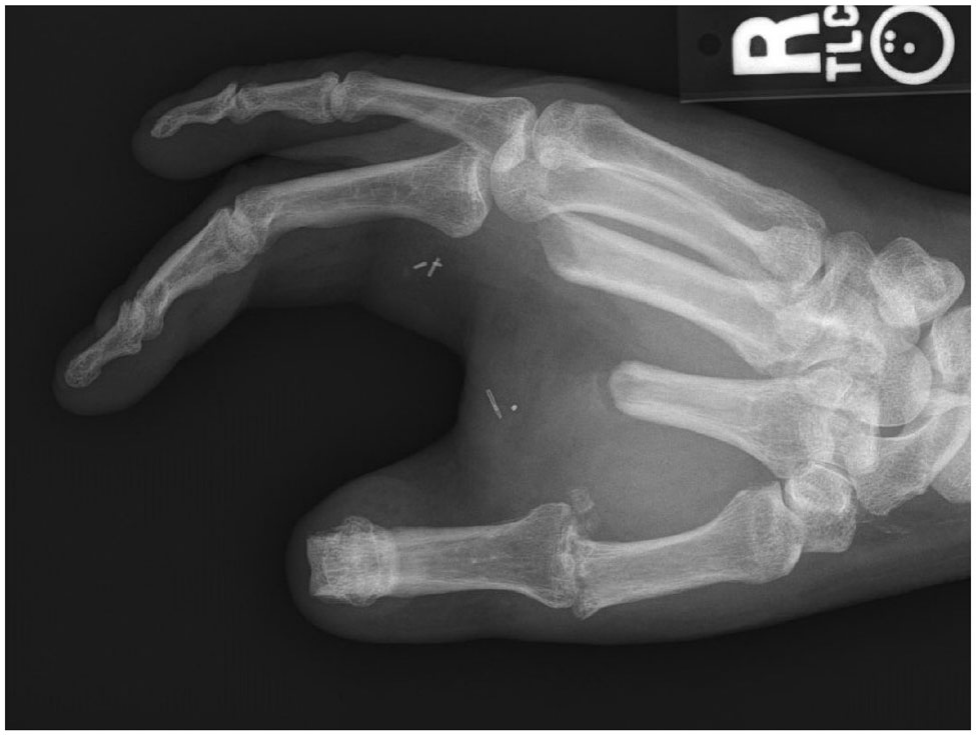
Nascent fusion between the thumb metacarpal and the middle finger proximal phalanx 3 months post-operative.

After intensive occupational therapy and tissue softening, at approximately 1.5 years after the initial injury, the patient was considered a good candidate for definitive reconstruction of the right hand, which was comparatively unusable (Figures 4 and 5). Discussion of options included prosthetics, transposition of the long finger, and toe-to-thumb transfer. Due to a complex social situation and poor function of the long finger flexors and extensors, the decision was made to transpose the long finger as a fixed/stable ‘post’ to the thumb position (Figures 6 and 7). The right long finger was transposed to the thumb position on the ulnar neurovascular pedicle, which was dissected to its origin at the palmar arch, and which was the only intact pedicle to the finger (Figure 8). The radial digital artery to the ring finger was ligated to allow further reach of the transposed long finger remnant, analogous to ligation of the radial digital artery to the long finger in a congenital case of index finger pollicization. The common digital nerve to the third webspace underwent an interfascicular dissection to also give further reach. Using a sagittal saw the right long finger was transected through the proximal phalanx close to the MP joint and part of the remaining metacarpal removed as a ray resection to deepen the webspace and improve prehension (Figure 9). The proximal phalanx was then fixed to the thumb metacarpal, which was beveled at 60 degrees and with moderate supination. Two 0.062 K-wires were placed retrograde into the thumb metacarpal and then driven obliquely into the new thumb proximal phalanx (Figure 10). Autologous bone grafting was performed to the site of bony union using metacarpal corticocancellous graft. At 3 months post-operatively he has adequate bony fusion and a 5-pound tip pinch strength (Figures 11–14).

## Discussion

For this injury pattern, which using an adaptation of Lister’s simple classification scheme would be described as total amputation with preservation of the carpometacarpal joint [12], there are multiple reconstructive options, including transposition, microsurgical toe transfer and metacarpal distraction. Advantages to toe transfer include strength, mobility and appearance. In a young, healthy, non-smoking patient with intact adjacent digits and high functional demand, toe transfer is considered the goal standard for thumb reconstruction at this level [13]. Distraction lengthening of the metacarpal is an another option in a patient with a compliant soft tissue envelope, with the advantage of native sensation. Given this patient’s age, extensive trauma, smoking status, predisposition to first webspace contracture, and uncertain ability to participate in rehabilitation, an ‘on-top’ plasty technique was chosen.

Transposition, or ‘on-top’ plasty, is a reliable, straightforward, and safe technique for thumb reconstruction, which crucially allows for native sensation. The technical details of this procedure are borne from the experiences of treating devastating hand trauma as well as congenital thumb absence, as many of same techniques and considerations are applicable to both ‘on-top-plasty’ and pollicization [14]. Pollicization is the cousin of the ‘on-top-plasty’, with the critical difference being that pollicization includes the transfer of all tendons and re-orientation of anatomic structures, which is often not achievable in the setting of mutilating hand trauma [13]. In both techniques, however, transposing the long finger to the thumb position has been described with relative paucity in the literature as compared to transposition or pollicization of the index finger. This technique was suggested initially by the French surgeon Guermonprez in 1887 and later refined by Tanzer and Littler in 1948 [7,15]. This reports highlights several critical steps specific to transposition of the long finger. First, ‘on-top-plasty’ with the long finger requires intraneural neurolysis and vascular dissection to the level of the palmar arch in order to reach the thumb position. Second, this case demonstrates that transfer of the long finger to the thumb position can be achieved with an ulnar neurovascular pedicle, which although described, is more technically challenging as it reduces the arc of rotation, and is employed only in the instance of injury to the radial digital artery [15]. Third, an important secondary maneuver in this operation is ray amputation of the residual index and/or long finger metacarpals in order to deepen an otherwise scarred or shallow first webspace, improving prehension. However, it is important not to excessively shorten the third metacarpal because the adductor pollicis originates on it. Therefore, metacarpal shortening of the long finger should only be performed to the extent required for a broad webspace that allows for adequate pinch.

The basic principles of this case harken to identification of that which makes a hand useful: at least two digits which can oppose with some power, one of which may be a stable post, and a cleft to allow for prehension [4]. On the right hand, neither opposition nor grasp was possible with the thumb amputated at the metacarpal level. A mutilated digit may better employed in thumb reconstruction than in trying to salvage it’s poor function as a finger. In this case, transposition of the long finger to the thumb position afforded a stable post for tip pinch, and combined with ray amputations of the residual index and long fingers, a wide cleft capable of accommodating objects, essential for restoration of a useful hand.
